# The Evolution of Diagnostic Techniques in the Paleopathology of Tuberculosis: A Scoping Review

**DOI:** 10.20411/pai.v8i1.597

**Published:** 2023-10-18

**Authors:** Veronica Papa, Francesco M. Galassi, Elena Varotto, Andrea Gori, Mauro Vaccarezza

**Affiliations:** 1 Forensic Anthropology, Paleopathology and Bioarchaeology (FAPAB) Research Center, Avola, Italy; 2 Department of Economics, Law, Cybersecurity, and Sports Sciences, University of Naples “Parthenope,” Naples, Italy; 3 School of Science, Engineering and Health, University of Naples “Parthenope,” Naples, Italy; 4 Department of Anthropology, Faculty of Biology and Environmental Protection, University of Lodz, 90-237, Lodz, Poland; 5 Archaeology, College of Humanities, Arts and Social Sciences, Flinders University, Adelaide, SA, Australia; 6 I Division of Infectious Diseases, “Luigi Sacco” Hospital, ASST Fatebenefratelli Sacco, Milan, Italy; Department of Pathophysiology and Transplantation, Centre for Multidisciplinary Research in Health Science (MACH), University of Milan, Milan, Italy; 7 Curtin Medical School, Faculty of Health Sciences, Curtin University, Bentley, Perth, 6102 Western Australia, Australia; 8 Curtin Health Innovation Research Institute (CHIRI), Faculty of Health Sciences, Curtin University, Bentley, Perth, 6102 Western Australia, Australia

**Keywords:** Tuberculosis, spondylodiscitis, ancient DNA, Mycobacterium Tuberculosis Complex (MTBC), human remains

## Abstract

Tuberculosis (TB) is an ancient chronic infectious disease that remains a global health concern. In human remains, the most common and characteristic clinical signs are the skeletal modifications involving the spine, such as in Pott's disease. Diagnosing TB in ancient human remains is challenging. Therefore, in this systematic review, the authors investigated the studies assessing molecular diagnosis of Pott's disease in ancient human remains with the intention to survey the literature, map the evidence, and identify gaps and future perspectives on TB in paleopathology. Our systematic review offers a full contextualization of the history of Pott's disease in ancient times. Our search strategy was performed between August 2022 and March 2023. The authors initially identified 340 records, and 74 studies were finally included and assessed for qualitative analysis. Due to non-specific clinical signs associated with TB, how best to diagnose tuberculosis in human remains still represents a central point. Nevertheless, ancient DNA (aDNA) analysis, lipid biomarkers, and spoligotyping might be extremely useful tools in the study of TB in human remains. Moreover, we propose the extraction and study of immune response genes involved in innate and adaptive immunity versus *Mycobacterium spp*. as an innovative and vastly overlooked approach in TB paleopathology. Complementary methodologies should be integrated to provide the best approach to the study of TB in human remains.

## INTRODUCTION

Tuberculosis (TB) is a disease that was identified in ancient times, and its skeletal evidence is primarily represented by the characteristic changes to the spine (kyphosis or gibbus) that result from Pott's disease. The eminent Greek physician Hippocrates (c. 460–c. 375 BC) — or, to be more precise, the body of work known as the “Hippocratic Corpus” — accurately described several characteristics of clinical tuberculosis [1] and offered a clear description of spinal kyphosis, linking to damage of other organs that can be thought of as associated with TB [2–4]. He also noted that patients had a poor prognosis if the spinal curvature was above the diaphragm and if the patient was a child or young adult. Many other ancient and historical texts report recognizable descriptions of TB, where the disease might be identified as phthisis, scrofula, King's Evil, lupus vulgaris, and even consumption [5]. TB became particularly prevalent during the industrial revolution because of the increasing population density and worsening living conditions. Its incidence slowly decreased in the 20th century, thanks to improved dietary intake, hygiene habits, novel vaccines, and chemotherapeutic drug therapy [6].

TB ranks among the top 15 causes of death worldwide, and it is the second leading cause of infectious disease death after COVID-19, ranking higher than HIV/AIDS [7–9]. Despite substantial efforts, TB has not been eradicated for several reasons, including its pathogenesis, which makes TB difficult to treat and contain, antibiotic resistance, and the susceptibility of immunocompromised populations due to human immunodeficiency virus (HIV), poverty, and malnutrition, as well as population mobility [5]. Indeed, according to the latest report from the World Health Organization, almost 10.6 million people fell ill with TB in 2021, with an estimated 1.6 million deaths globally [6, 7]. Hence, despite slowly decreasing its incidence, TB remains a critical infectious disease worldwide.

The main causative organism of TB is *Mycobacterium tuberculosis*, a strictly aerobic bacterium, which is a member of the *M. tuberculosis* complex (MTBC) discovered by Robert Koch (1843-1910) in 1882. The MBTC includes *M. bovis* and *M. africanum, M. canettii, M. pinnipedii, M. microti, M. caprae, M. mungi*, and *M. orygis*. Different hypotheses regarding the start and subsequent evolution of tuberculosis have been proposed over the years, making it a controversial topic that still needs to be fully understood and further investigated. According to paleopathological evidence, in the late 1990s, it was thought that humans acquired tuberculosis from animals with the advance of domestication and MTBC's persistence and spread were related to the density of human populations and migration [10–13]. Therefore, it was thought that TB had a zoonotic origin and was acquired by humans from cattle during the Neolithic revolution [14–16]. It seemed likely and scientifically sound to postulate that Columbus and subsequent European colonization introduced TB to the New World (the Americas), although granulomas have been detected in pre-Columbian mummified tissue [17].

Of note, a more recent paleogenetic hypothesis suggests that the bovine form of the disease is derived from human strains [10]. Furthermore, detailed anatomical studies have been performed on recent historical skeletal collections to assess and agree on diagnostic criteria based on skeletal modifications [2]. Accordingly, some skeletal changes, such as rib periostitis (surface shape modifications caused by new bone formation), were non-specific clinical signs significantly associated with individuals diagnosed with clinical TB [18, 19]. In addition, paleohistological techniques allowed the identification of more narrow changes associated with TB in calcified and non-calcified tissues. Finally, signs of TB might be detected as granulomas in the lungs and other organs [20]. However, skeletal tuberculosis is likely to occur at a low rate in patients diagnosed with pulmonary TB, leading to the assumption that the incidence of TB in ancient human remains is extremely underestimated [2]. As far as ancient Egyptian mummies are concerned, an early identification of the presence of tubercular changes was related to the collapse of the vertebral body, particularly in the ventral-central portion of the vertebral bodies, which is typical of Pott's disease, the peculiar spinal modification first described by the English surgeon Sir Percivall Pott (1714-1788). Subsequently, the mummy of Nesperehan was investigated by Sir Marc Armand Ruffer (1859-1917) who linked skeletal evidence with a large abscess of the psoas muscle [21]. Later, in the 1960s, Morse extended this methodological approach by highlighting extensive skeletal lesions and associated kyphosis in a dozen mummies [15]. A further step forward was made by Zimmerman (1977), who, by analysing human remains in the tomb of Nebwenenenf (a priest under Ramses the Great, 1279-1212 BC), combined the microscopic visualization of bacilli in rehydrated vertebral bodies with the evidence of blood in the remnants of trachea and lungs (highlighting a pulmonary haemorrhage) [22]. Finally, in 2001, Zink and colleagues [23] announced that “as a positive molecular reaction was observed in most of the typical cases of skeletal tuberculosis, in about one-third of non-specific, but probable tuberculous osseous changes and, surprisingly, in about one-seventh of unremarkable samples, this suggests that infection with *M. tuberculosis* was relatively frequent in ancient Egypt.” [24]. Indeed, the mycobacteria grouped in the *Mycobacterium tuberculosis* complex are highly conserved. Their gene sequences showed a high degree of conservation, being characterized by more than 99% similarity at the DNA level [24–27]. Moreover, they show widely different host tropisms, phenotypes, and pathogenicity features, resulting in variable disease manifestations, immunological responses, and eventually, the frequency of drug resistance and ability to escape vaccination [28–33].

Although this hypothesis is still a matter of debate [34, 35], genomic sequencing provided evidence that the progenitor of *M. tuberculosis* strains already represented a human pathogen when *M. africanum* and *M. bovis* separated from the *M. tuberculosis* lineage. Furthermore, Gordon and co-workers, as well as Brosch and co-workers [10, 34] demonstrated that the genome of *M. bovis* is smaller than that of *M. tuberculosis*. Therefore, it seems likely that *M. bovis* is the final member of a separate lineage illustrated by *M. africanum, M. microti*, and *M. bovis*, which derived from the progenitor of *M. tuberculosis* isolates. Of note, TB susceptibility remained unclear and elusive until recently, when a FokI polymorphism in the vitamin D receptor gene was associated with increased susceptibility to spinal tuberculosis [36]. Moreover, autosomal-recessive interleukin-12 receptor b1 (IL-12Rb1) and tyrosine kinase 2 (TYK2) deficiencies have been correlated with a serious form of TB [37, 38]. Nevertheless, homozygosis predisposition to TB was recognized in 2018 and described as the homozygotic trait for the TYK2/P1104A polymorphism. Furthermore, P1104A carriers were found to be at higher risk of having clinical forms of TB; further analysis of 1013 ancient human genomes demonstrated that the P1104A variant originated in the common ancestors of West Eurasians 30 000 years ago [39, 40].

Regardless of the type of techniques used for its diagnosis, a thorough understanding of TB, as well as *M. tuberculosis* evolution and genetics, might lead to increased knowledge of the organism's pathogenesis and subsequently provide better treatments and control measures. Moreover, specific and highly associated TB morphological and clinical signs need to be matched to molecular analysis to provide an accurate and reliable diagnosis of TB in ancient human remains. Therefore, the aim of this study was to survey the literature, map the evidence, and identify gaps and future perspectives in the molecular paleopathology of TB.

## MATERIALS AND METHODS

According to the 5-stage protocol by Arksey and O'Malley [41], Levac and coworkers [42], and Westphaln et al [43], a scoping review was performed between August 2022 and March 2023. The steps have been assessed in the identification of the research question, the identification of relevant studies, the selection of specific studies, and finally, reporting of the results. The included records were then screened, and a thematic analysis was performed. Since this study did not include human material (cells, tissues, organs, patients, or others) but was only limited to already published research, approval from an Ethical Committee was not required. In addition, the manuscript was not classified as eligible for an ethical review.

### Search Strategy

The search strategy was carried out in the time range detailed above. It was based on key search terms in PubMed (US National Library of Medicine, National Institutes of Health, Bethesda, MD), Biomed Central (BioMed Central Ltd., Springer Nature, London, UK), Scopus (Elsevier B.V., Amsterdam, the Netherlands), and Google Scholar (Google Inc., Mountain View, CA) search engines. The search strategy was designed by V.P. and F.M.G. and validated by one of the senior authors (M.V.).

### Eligibility Criteria

A literature search, including the terms, “MBTC OR tuberculosis AND human remains AND aDNA”, was carried out in the above-mentioned search engines.

Inclusion criteria encompassed research articles published in English-language and peer-reviewed journals describing Pott's disease in ancient human remains and focused on molecular diagnosis. Keywords linked to these terms were identified. Furthermore, the authors analyzed the reference lists of articles identified through this search strategy and selected additional publications that they deemed relevant.

### Study Selection and Data Extraction

Titles, abstracts, keywords, and full texts were reviewed by 2 authors (V.P. and F.M.G.). Conflicts between reviewers were discussed until a consensus was reached, and the senior author (M.V.) was involved if needed. A total of 340 records were initially identified. After duplicate removal, a total of 139 records were further considered.

Abstracts, keywords, and the complete reference lists were analyzed for all articles. Only items in which the abstract unequivocally discussed the topic were included. Therefore, 66 records were excluded with reason, and 74 were finally assessed for full-text screening and further analysis.

The Preferred Reporting Items for Systematic reviews and Meta-Analysis (PRISMA) flow-chart was utilized for the reporting of findings [44–47], and it is available in the Results section (see below). The records included in this study are listed in Supplemental Tables 1 and 2. All the authors agreed on the final number of studies included.

### Qualitative Analysis

According to the Grounded Theory methodology [48, 49], the authors broadly discussed the basis of their query before formulating the research questions: “What is the status of the paleopathology of tuberculosis? Is molecular diagnosis a reliable tool to unveil disputed cases?”

The inclusion of records in the qualitative synthesis was performed using the online Research Screener machine learning tool for systematic reviews [50]. The sample in this qualitative analysis was represented by the records assessing the use of molecular techniques in diagnosing TB in human remains.

Initially, the data set imported into the Research Screener machine learning tool was represented by the 74 records assessed for qualitative analysis. Credibility and reliability were ensured by debriefing and triangulation.

After exhaustive discussion among team members, 7 seed articles were identified [6, 51–56]. According to the instructions, the library created with the 74 records assessed for qualitative analysis was uploaded to the tool to be further analyzed. Moreover, no abstract from the library counted less than 100 words and no missing abstracts were automatically removed by the tool. Therefore, a final set of 74 abstracts was further analyzed. Two reviewers (V.P. and E.V.), and an external expert who has full experience in qualitative research independently flagged the abstract. Conflicts were discussed between reviewers and managed accordingly. The senior author (M.V.) was involved if needed. The final qualitative synthesis included 74 records. Subsequently, members of the team (V.P., E.V., and F.M.G.) independently coded and categorized the data into themes and subthemes, which were debated on a regular basis.

Open codes were developed, and themes and subthemes were generated using the trial version of NVivo qualitative data analysis software package (QSR International Pty, Ltd., Melbourne, VIC, Australia) [57, 58]. These themes were reviewed by all authors to ensure they were fully consistent with the research question. Any further discrepancy was discussed to minimize bias.

## RESULTS AND DISCUSSION

The authors discussed broadly the foundations of their query; 2 members of the team (V.P., F.M.G.) and an external expert independently extracted the key topics, which were discussed on a regular basis.

**Figure 1.**
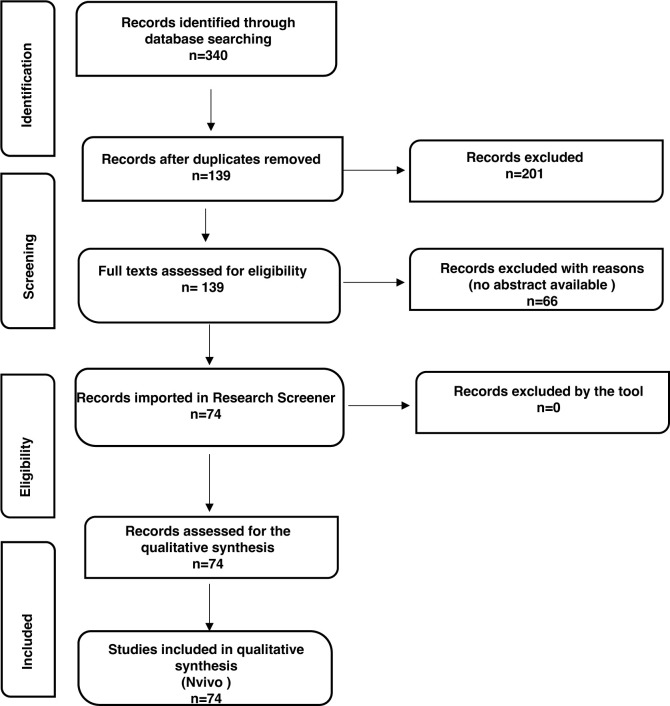
**Flowchart of the Reporting items for the systematic review adapted from the Preferred reporting items for systematic reviews (PRISMA) statement [44].** Researchers initially identified 340 records; 74 were included and further processed using the open, online Research Screener machine learning tool (Research Screener, 2021) and evaluated for the qualitative synthesis using the NVivo qualitative data analysis software (QSR International Pty, Ltd., Melbourne, VIC, Australia).

All authors reviewed these pools to ensure they were entirely consistent with the research question and identified themes that are examined in the following paragraphs. Any further discrepancy was discussed to minimize bias. The flowchart of the *Reporting items for the systematic reviews* (adapted from the *Preferred reporting items for systematic reviews (PRISMA)* statement) is reported below (Figure 1).

### Qualitative Synthesis

Tuberculosis (TB) is an ancient chronic infectious disease predominantly affecting the lungs and remains a global health concern. The most common and characteristic clinical signs in human remains are the skeletal changes involving the spine, such as in Pott's disease, which accounts for more than 40% of all cases of skeletal tuberculosis. Moreover, other than ankylosis, spondylitis, and other vertebral lesions that might cause vertebral collapse, non-specific clinical signs, including extraspinal unifocal lytic lesions, single joint ankylosis, new bone formation, periostitis, and serpens endocrania symmetrica, have been highly associated with — albeit non-specific to — TB.

**Figure 2.**
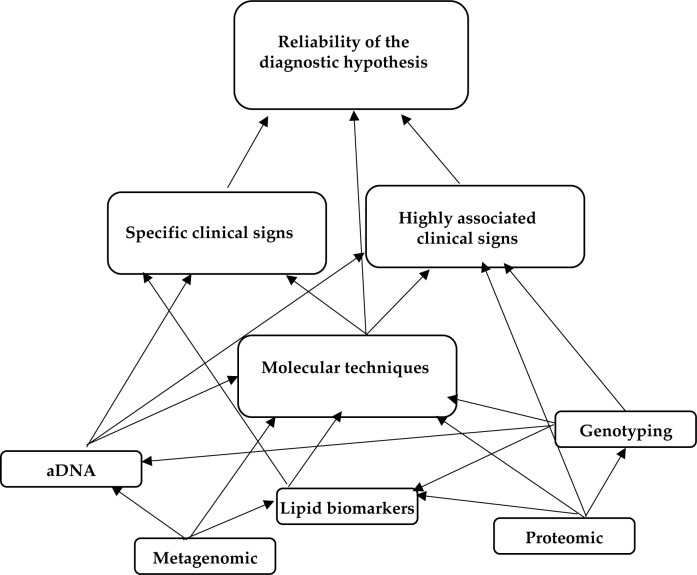
**Node diagrams based on NVivo qualitative data analysis software output (QSR International Pty, Ltd., Melbourne, VIC, Australia).** The larger boxes have been used to identify themes, the smaller to define subthemes.

Therefore, one main theme was generated from the results: exploring the reliability of the diagnosis of tuberculosis in ancient human remains. Subthemes covered numerous aspects, including the way in which molecular techniques, aDNA analysis, and lipid biomarkers, could help in assessing the diagnosis. Also, subthemes were identified about the impact that genotyping, proteomics, and metagenomics could have in unveiling hidden cases of MBTC infections in human remains (Figure 2). Themes and subthemes are discussed in the following paragraphs.

### Established and Emerging Techniques for Molecular Studies

With the introduction of the polymerase chain reaction (PCR), genetic studies on ancient human remains became extremely popular and widely used, although with an initial lack of adequate measures and protocols for safely handling aDNA, as well as preventing contamination with modern DNA [59–61]. Protocols and safety measures were implemented and modified according to the increased comprehension of the sources of contamination [62–64]. Tuberculosis was the first infectious disease to be successfully detected by PCR in skeletal remains and mummified tissue [65–67]. More recently, Wooding and co-workers [68] evaluated the presence of aDNA from MTBC complexes in both human and faunal assemblages from a multiperiod site in the United Kingdom, and highlighted the importance of DNA degradation in ancient human remains as well as the importance of evaluating the evidence for zoonotic diseases at sites where human and faunal conglomerations are recovered. Although whole genome amplification (WGA) has been suggested as a reliable method to study aDNA of members of the MTBC, Forst and colleagues recently disproved this hypothesis and concluded that WGA does not supply any advantage in studies of MTBC aDNA [69]. Following the complete *M. tuberculosis* genome sequencing in 1998, several comparative genomics studies demonstrated that members of the MTBC have likely evolved thanks to mutations in their genome, mainly single nucleotide polymorphisms (SNP), insertions, and deletions [34, 70, 71]. A key feature of the bacterial genome, the mobile genetic elements (MGEs), can move through the genome and eventually cause genomic rearrangement and polymorphism. The most common polymorphism in *M. tuberculosis* is the Insertion Sequence 6110 (IS6110) [72–74], which is usually present within the genome in multiple copies. This polymorphism is extremely variable in terms of the number of copies and its position within the bacterial cell. Therefore, the insertion sequence IS6110 has become the preferred locus for detecting tubercle bacilli and has been widely used as a genotypic marker in epidemiological studies [75, 76]. More recently, Borówka and co-workers demonstrated the presence of DNA from MTBC in ancient human remains by revealing skeletal lesions consistent with vertebral tuberculosis by gene sequencing [77]. Moreover, Harkins et al designed 4 quantitative PCR (qPCR) assays to target regions within the MTBC to examine 133 human skeletal samples with skeletal lesions consistent with clinical signs of chronic disseminated tuberculosis [55].

Molecular studies confirmed TB in ancient human remains from necropoles in Ancient Egypt [78], Asia [79, 80], America [81–84], and Europe [77, 85–100]. These authors reported aDNA extracted and analyzed and positively tested for MBTC, both from vertebral lesions and highly associated lesions. Some of the included records further analyzed and discussed these findings in available reviews [6, 28, 51–54, 56, 101–110].

Other than aDNA analysis, human remains might be tested for MTBC by analyzing mycolic acids. These molecules are strain-specific long-chain fatty acids associated with the cell envelope that helps with bacterial resistance to environmental stresses. Moreover, their distribution is different among mycobacteria. First identified by Gernaey and co-workers in 2001 [107], they represent a specific biomarker of the bacterial cell wall, offer independent confirmation of TB [35, 79, 80, 85, 90, 92, 101, 107, 110], and might be a useful method to verify the diagnosis of tuberculosis. Nevertheless, false positives can be caused by environmental contamination when applying mycolic acid techniques [77]. More recently, mycocerosic acids and other waxes of the bacterial envelope have been demonstrated to be more stable than mycolic acids, and might be useful lipid biomarkers, in the diagnosis of TB [84, 92, 93, 103, 111]. Redman and co-workers [111] analyzed the mycocerosate profile of 49 individuals from the 1837–1936 Coimbra collection: almost 50% of the investigated individuals had clinical records identifying tuberculosis as the possible cause of death. Moreover, the authors reported a 72% correlation between the presence of the analyzed biomarkers and the individuals reported to be likely to have died from TB.

### TB Paleopathology in the Light of New Molecular Techniques: From Bones to Molecules

Among others, Zink and colleagues [112] and Baron et al [113] demonstrated that the presence of MBTC DNA in ancient human remains might not correlate with the clinical appearance of the bones from which that DNA had been extracted, being apparently normal bone samples positive for MTBC aDNA. Therefore, these authors demonstrated that *M. tuberculosis* infection could be diagnosed by molecular techniques in the absence of lesions and hypothesized that the infection spread to the bones through the bloodstream. Consistent with those findings, in 2002, Spiegelman and co-workers [114] re-examined and confirmed the presence of MTBC aDNA in previously reported human remains [52]. In the same year, Konomi and colleagues [115] demonstrated the presence of MTBC DNA in mummified genital tissue samples from 12 pre-Columbian Andean mummies exhibited in the American Museum of Natural History in New York.

In contrast, Mays et al [116] examined the rib lesions in 7 skeletons from a rural English Medieval necropolis. The authors aimed to establish if these lesions regularly correlated with tuberculous infection were detected by PCR. Unfortunately, they were unable to provide solid proof for any regular association between visceral surface rib lesions and the presence of *M. tuberculosis* complex DNA and suggested that the absence of clinical signs does not rule out the detection of tuberculous biomarkers.

### Current Status and Future Perspectives

As previously stated, the high degree of conservation within their genome, as well as the presence of strain-specific variable sequences and lipids biomarkers, allows aDNA and chemical analysis to be powerful tools to distinguish between different strains and lineages of the MTBC and to study their evolution and pathogenic features [117, 118]. Moreover, the results from aDNA analysis might be unreliable due to environmental contamination while lipids analysis is performed by fluorescence High-Performance Liquid Chromatography (HPLC) that does not involve the amplification of target molecules. As a conservative method, it has the advantage of not being subject to contamination and does not compromise the integrity of the analyzed sample.

Molecular methods primarily involve the analysis of repeated sequences, deletions, and SNPs. In this regard, although the IS6110 region is the preferred target sequence to assess aDNA, other portions of the *M. tuberculosis* genome have been assessed. In 1997, Kamerbeek [119] and colleagues proposed spoligotyping as an innovative and valuable method to easily and simultaneously detect and type *M. tuberculosis* in clinical specimens. The method is based on polymorphism of the chromosomal direct repeat (DR) locus. This locus, which contains the insertion element IS6110, is characterized by multiple and short repeated sequences that are extremely conserved among *M. tuberculosis* strains. These DR sequences probably originated from recombination between DR sequences as well as from rearrangements caused by the insertion of IS6110. Furthermore, the DR sequences are interspersed within the genome with nonrepetitive sequences resulting in 34 to 41 bp long strain-dependent spacers. Therefore, DR sequences represent a potential target for *in vitro* PCR amplification while the spacers' variability became a suitable target to differentiate *M. tuberculosis* strains [120]. Zink and co-workers [121] used spoligotyping to reveal and characterize MBTC aDNA in mummified soft tissue samples from 85 ancient Egyptian mummies and reported for the first time a case of human TB infection caused by *M. africanum*. Finally, the SNP analysis might be a valuable tool to study the past MTBC lineages and their evolution. In 1997, Sreevatsan et al [25] first described 2 SNPs that occur at high frequency in the genes encoding catalase peroxidase and the A subunit of gyrase and were able to identify 3 genetic groups of *M. tuberculosis*, thereby proposing an evolutionary pathway for MTBC. Later, Brosch and colleagues [10] showed that the SNP types identified by Sreevatsan et al took place in a lineage of *M. tuberculosis* strains that had already lost *M. tuberculosis*-specific deletion 1 (TbD1). Therefore, the authors defined ancestral or *modern* MBTC strains according to the presence or absence of TbD1. More recently, Fletcher et al [88] analyzed *M. tuberculosis* aDNA amplified from naturally mummified tissues from 18^th^ and 19^th^ centuries and reported that some individuals were characterized by katG463/gyrA95 SNPs as well as the TbD1. Therefore, the authors clearly demonstrate that this deletion occurred in the lineage of *M. tuberculosis* before the 18th century and suggested that this mutated strain was the cause of the subsequent increased incidence of TB in the 18th century. These findings were definitively confirmed by Hershkovitz and colleagues in 2008 [90].

More recently, Boros-Major and colleagues [100], as well as Hajdu and co-workers [89], analyzed mycobacterial protein from ancient human remains displaying skeletal signs of Pott's disease using mass spectroscopy demonstrating that proteomic analyses might be a valuable tool in supporting the morphological diagnosis and might help to understand the prehistoric epidemiology and evolution of these pathogens.

Differentiating between genuine human pathogens and closely related environmental counterparts can pose a challenge, particularly in the identification of *Mycobacterium* species through repetitive elements. These mobile elements resemble those present in other mycobacterial agents or soil microorganisms, complicating the process. PCR assays are frequently utilized as a costly yet efficient solution to swiftly narrow down the most promising samples for deeper and more targeted shotgun sequencing. The emergence of high-throughput sequencing technology led to a transition from basic identification of pathogens in specific locations and moments to the more advanced genome-level reconstruction and analysis. This allowed for a shift towards hypothesis-driven research conducted within evolutionary frameworks [122].

The use of metagenomics and whole-genome sequencing has had a significant impact on our understanding of infectious diseases in the past [123]. These techniques have allowed researchers to identify ancient pathogens and study their genetic makeup. This has enabled researchers to reconstruct the evolutionary history of pathogens and trace their spread across different populations [123].

Of note, new techniques could allow a more extensive and deeper analysis of host immune genes in paleopathology. Host immune gene analysis carries the ability to significantly impact our understanding of infectious diseases in the past. This technique has allowed researchers to study how ancient humans responded to different pathogens [124]. By studying the genetic material that codes for the immune system, researchers can identify which genes were under positive selection in response to different pathogens, and even trace the host-bacterium relation from the evolutionary point of view.

One example of the use of metagenomics and whole-genome sequencing in paleopathology is the study of *Yersinia pestis*, the bacterium responsible for the Black Death. Researchers were able to extract DNA from the teeth of individuals who died during the Black Death and sequence the entire genome of *Y. pestis*. This step allowed researchers to reconstruct the evolutionary history of the bacterium and trace its spread across Europe [124, 125]. These results underscore the role of natural selection to the present-time human susceptibility towards chronic inflammation and autoimmune disease. Selection for defence in the presence of pathogenic microbes such as *Y. pestis* [124] may be counterbalanced against the costs of immune disorders, resulting in a long-term signature of balancing selection [126, 127]. The same scenario could be easily applied in the context of an immune-system-TB relation in paleopathology studies, helping us to better delineate the selection of protective immune responses versus *Mycobacterium spp*. as well as the evolutionary *price* in terms of genetic predisposition to immune disorders.

## CONCLUSIONS

Madhukar Pai (McGill International TB Centre, Montreal, QC, Canada) reported in *The Lancet Microbe*, “Right now, improving tuberculosis case detection is urgent. All countries have scaled up molecular testing and genetic sequencing capacity for COVID-19, and this expanded capacity could be used for tuberculosis testing” [9]. Moreover, Henneberg and coworkers [128] defined tuberculosis as a widespread chronic infectious disease that represents an example of the co-evolution of host and pathogen, whose paleopathology has been well documented. Judging from the available perused literature, it appears that the widest possible combination of complementary methodologies should be implemented to diagnose ancient mycobacterial disease in order to attain highly reliable phenotypic and genotypic levels of evidence. For skeletal material, significant expertise has been developed in the past decades in recognizing characteristic bone changes linked to tubercular infection [129]. The precise diagnosis of tuberculosis in the contemporary scientific setting needs recognition of pivotal biomarkers for the causative agent *M. tuberculosis*. The new frontier of whole-genome sequencing and metagenomics is the way to dissect the relationship between a given pathogen and the immune system, giving us the possibility to better understand the trajectory of TB versus our immune response, starting from early interaction (such as in ancient specimens) to our contemporary times.

While continuing to enrich the global record of paleopathological cases, further research should be aimed at standardizing a diagnostic protocol, potentially complementing older methods with newer ones while reassessing previously published cases, and at exploring the evolution of this disease and its interaction with human life on this planet.

## SUPPLEMENTARY DATA

Supplementary materials are available at the *Pathogens and Immunity* website. Supplementary data may be provided by the authors to benefit the reader. Supplementary data are not copyedited and are the sole responsibility of the authors. Questions or comments related to supplementary materials should be addressed to the corresponding author.
