# Supplementary material for: The Evolution of Diagnostic Techniques in the Paleopathology of Tuberculosis: A Scoping Review
Source: Pathog Immun. 2023 Oct 18;8(1):93–116. doi: 10.20411/pai.v8i1.597 (PMC10603826; doi:10.20411/pai.v8i1.597)
Supplement: Supplemental Tables 1 and 2 [file pai-8-093-s01.pdf]

PATHOGENS AND IMMUNITY
IN ASSOCIATION WITH CASE WESTERN RESERVE UNIVERSITY AND THE RICHARD J. FASENMYER FOUNDATION

SUPPLEMENTARY TABLES 1 and 2

Supplementary Table 1. List of the Included Records (Authors A-L).

Study details	Title	Type of study	Keywords
Anastasiou & Mitchel *(2013) Gene*	Palaeopathology and genes: Investigating the genetics of infectious diseases in excavated human skeletal remains and mummies from past populations	Review	Ancient disease; Ancient DNA; Mummies; Palaeomicrobiology; Palaeoparasitology; Palaeopathology
Baker et al. (2015) *Tuberculosis*	Human tuberculosis predates domestication in ancient Syria	Original article	Paleopathology of TB; early neolithic; PPNB; agriculture cradle; domestication; lipid biomarkers; ancient DNA
Baker et al. (2017) *Paléorient*	Prehistory of human tuberculosis: Earliest evidence from the onset of animal husbandry in the near east	Original article	Syria; neolithic; domestication; early PPNB; Tuberculosis; Paleopathology
Baron et al. (1996) *J Archaeol Sci*	*Mycobacterium Tuberculosis* Complex DNA in Ancient Human Bones	Original article	Not available
Borówka et al. (2019) *Gigascience*	Screening methods for detection of ancient *Mycobacterium tuberculosis* complex fingerprints in next-generation sequencing data derived from skeletal samples	Original article	Ancient DNA; aTB; ancient tuberculosis; NGS
Bouwman et al. (2012) *PNAS*	Genotype of a historic strain of*Mycobacterium tuberculosis*	Original article	Biomolecular archaeology; paleopathology
Bianucci et al. (2012) *Int J Paleopath*	Eleonora of Toledo (1522–1562): Evidence for tuberculosis and leishmaniasis co-infection in Renaissance Italy	Case study	*Mycobacterium tuberculosis* complex; visceral leishmaniasis; ancient DNA; Renaissance; Italy
Boros-Major et al. (2011) *J Archaeol Sci*	New perspectives in biomolecular paleopathology of ancient tuberculosis: a proteomic approach	Original article	Biomarker discovery; biomolecular archaeology; MALDI TOF mass spectrometry; *Mycobacterium tuberculosis*; paleopathology; proteomics
Brosch et al. (2002) *Proc Natl Acad Sci U S A*	A New Evolutionary Scenario for the *Mycobacterium tuberculosis* Complex	Original article	Not available
Cole et al. (1998) *Nature*	Deciphering the Biology of *Mycobacterium tuberculosis* from the Complete Genome Sequence	Letter to the editor	Not available
Crubezy et al. (1998) *Anthropologie*	Identification of Mycobacterium DNA in an Egyptian Pott's disease of 5,400 years old	Original article	Ancient DNA; paleopathology; tuberculosis; anthropology
Darling & Donoghue (2014) *Mem Inst Oswaldo Cruz.*	Insights from paleomicrobiology into the indigenous peoples of pre-colonial America - a review	Review	Ancient DNA; *Helicobacter pylori*; *Mycobacterium tuberculosis*; *Trypanosoma cruzi*; *Coccidioides immitis*
Dawson et al. (2012) *Int J Paleopathol*	Childhood Tuberculosis: A Probable Case from Late Mediaeval Somerset, England	Case Study	Not available
Donoghue et al. (2004) *Lancet*	Tuberculosis: from prehistory to Robert Koch, as revealed by ancient DNA	Review	Not available

Donoghue (2009) *Microbes Infect*	Human tuberculosis--an ancient disease, as elucidated by ancient microbial biomolecules	Review	Ancient DNA; Lipid biomarkers; *Mycobacterium tuberculosis*; Paleogenetics; Paleomicrobiology
Donoghue (2011) *Clin Microbiol Infect*	Insights gained from palaeomicrobiology into ancient and modern tuberculosis	Review	Ancient DNA, lipid biomarkers, *Mycobacterium tuberculosis*, paleomicrobiology, paleopathology
Donoghue et al. (2015) *Tuberculosis*	Ancient DNA analysis - An established technique in charting the evolution of tuberculosis and leprosy	Review	Ancient DNA
Donoghue (2016) *Microbiol Spectrum*	Paleomicrobiology of human tuberculosis	Review	Not available
Donoghue (2017) *Int J Infect Dis*	Insights gained from ancient biomolecules into past and present tuberculosis—a personal perspective	Review	Ancient DNA (aDNA); Bacterial cell wall lipids; Evolution
Donoghue et al. (2017) *Diversity*	Positive Diagnosis of Ancient Leprosy and Tuberculosis Using Ancient DNA and Lipid Biomarkers	Review	aDNA; cell wall lipids; evolution; genotyping; Mycobacterium leprae; Mycobacterium tuberculosis; palaeopathology
Dutour (2016) *Microbiol Spectr*	Paleopathology of Human Infections: Old Bones, Antique Books, Ancient and Modern Molecules	Perspective	Not available
Fletcher et al. (2003) *Am J Physical Anthropol*	Widespread Occurrence of *Mycobacterium tuberculosis* DNA From 18th–19th Century Hungarians	Original article	*Mycobacterium tuberculosis*; ancient DNA; PCR; paleomicrobiology; history of disease
Forst & Brown (2016) *PLoS ONE*	Inability of 'Whole Genome Amplification' to Improve Success Rates for the Biomolecular Detection of Tuberculosis in Archaeological Samples	Original article	Not available
Gad et al. (2021) *Hum Mol Genet*	Insights from Ancient DNA Analysis of Egyptian Human Mummies: Clues to Disease and Kinship	Review	Not available
Garnier et al. (2003) *Proc Natl Acad Sci U S A*	The Complete Genome Sequence of *Mycobacterium bovis*	Original article	Not available
Gernaey et al. (2001) *Tuberculosis (Edinb).*	Mycolic Acids and Ancient DNA Confirm an Osteological Diagnosis of Tuberculosis.	Original article	Not available
Gordon et al. (1999) *Microbiology*	New Insertion Sequences and a Novel Repeated Sequence in the Genome of *Mycobacterium tuberculosis* H37Rv	Original article	*Mycobacterium tuberculosis*; insertion sequence; prophage
Gordon et al. (2001) *Tuberculosis*	Genomics of *Mycobacterium bovis*	Original article	Not available
Groenen et al. (1993) *Mol Microbiol*	Nature of DNA Polymorphism in the Direct Repeat Cluster of *Mycobacterium tuberculosis*; Application for Strain Differentiation by a Novel Typing Method	Original article	Not available
Guichón et al.(2015) *Int J Palaeopath*	Pre-Columbian tuberculosis in Tierra del Fuego? Discussion of the paleopathological and molecular evidence	Case study	Tuberculosis; ancient DNA; tierra del Fuego; bioarchaeology

Hajdu et al. (2012) *Mem Inst Oswaldo Cruz*	Bone tuberculosis in Roman Period Pannonia (western Hungary)	Original article	Spinal tuberculosis; mycolic acid analysis; proteomic analysis
Hajdu et al. (2012) *Spine*	A case of spinal tuberculosis from the Middle Ages in Transylvania (Romania)	Case study	Not available
Harkins et al. (2015) *Phil Trans R Soc B*	Screening ancient tuberculosis with qPCR: challenges and opportunities	Original article	Ancient DNA; tuberculosis; bioarchaeology; qPCR
Hershkovitz et al. (2008) *PLoS ONE*	Detection and Molecular Characterization of 9000-Year-Old *Mycobacterium tuberculosis* from a Neolithic Settlement in the Eastern Mediterranean	Original article	Not available
Hershkovitz et al. (2015) *Tuberculosis*	Tuberculosis origin: The Neolithic scenario	Original article	Ancient DNA; neolithic; origin of tuberculosis; paleopathology
Jaeger et al. (2012) *Infect Genet Evol*	*Mycobacterium tuberculosis* Complex Detection in Human Remains: Tuberculosis Spread since the 17th Century in Rio de Janeiro, Brazil	Original article	Tuberculosis; ancient DNA; human mtDNA
Jaeger et al. (2013) *Emerg Infect Dis*	*Mycobacterium tuberculosis* Complex in Remains of 18th–19th Century Slaves, Brazil	Letter to the editor	Not available
Kalová et al. (2019) *Int J Paleopath*	Serious chronic disease of the cervical spine and trauma in a young female from the middle ages (Czech Republic)	Original article	Paleopathology; tuberculosis; osteomyelitis; mycosis; trauma; fracture; Pohansko; early middle ages
Kamerbeek et al. (1997) *J Clin Microbiol*	Simultaneous Detection and Strain Differentiation of *Mycobacterium tuberculosis* for Diagnosis and Epidemiology	Original article	Not available
Konomi et al. (2002) *J Clin Microbiol*	Detection of Mycobacterial DNA in Andean Mummies	Original article	Not available
Lee et al. (2015) *Tuberculosis (Edinb)*	Lipid Biomarkers Provide Evolutionary Signposts for the Oldest Known Cases of Tuberculosis	Original article	Ancient tuberculosis; lipids; biomarkers
Luna et al. (2020) *Tuberculosis*	Oldest evidence of tuberculosis in Argentina: A multidisciplinary investigation in an adult male skeleton from Saujil; Tinogasta; Catamarca (905–1030 CE)	Original article	Infectious diseases; pre-hispanic populations; paleopathology; Pott's disease; *M. tuberculosis* complex; tuberculous spondylitis

The table details the main features of the included records: authors, type of study and keywords. Abbreviations: PPNB, Pre-pottery Neolithic B; NGS, next-generation sequencing; PCR, polymerase chain reaction; qPCR, quantitative polymerase chain reaction; aDNA, ancient DNA; IS, insertion sequences.

Supplementary Table 2. List of the Included Records (Authors M-Z).

Study details	Title	Type of study	Keywords
Masson et al. (2013) *PLoS ONE*	Osteological and Biomolecular Evidence of a 7000-Year-Old Case of Hypertrophic Pulmonary Osteopathy Secondary to Tuberculosis from Neolithic Hungary	Original article	Not available
Masson et al. (2015) *Tuberculosis*	7000 year-old tuberculosis cases from Hungary - Osteological and biomolecular evidence	Original article	Tuberculosis; neolithic; Hungary; aDNA; lipid biomarkers
Mays et al. (2002) *Am J Phys Anthropol*	Investigation of the Link between Visceral Surface Rib Lesions and Tuberculosis in a Medieval Skeletal Series from England Using Ancient DNA	Original article	Palaeopathology; Wharram Percy; ancient biomolecules
McEvoy et at. (2007) *Tuberculosis*	The Role of IS6110 in the Evolution of *Mycobacterium tuberculosis*	Review	*Mycobacterium tuberculosis*; IS6110; transposon; evolution
Minnikin et al. (2015) *Tuberculosis (Edinb)*	Ancient Mycobacterial Lipids: Key Reference Biomarkers in Charting the Evolution of Tuberculosis	Review	Tuberculosis; evolution; lipids;biomarkers; zoonosis
Muller et al. (2014) *Am J Physical Anthropol*	Biomolecular Identification of Ancient *Mycobacterium tuberculosis* Complex DNA in Human Remains From Britain and Continental Europe	Original article	Infectious disease; IS elements; PCR; skeletal pathology; visceral surface new bone
Muller et al. (2015) *Sci Technol Archaeol Res*	Complications in the Study of Ancient Tuberculosis: Non-Specificity of IS6110 PCRs	Original article	Ancient DNA; Britain; IS6110; *Mycobacterium tuberculosis* complex; roman period, tuberculosis
Muller et al. (2016) *J Archaeol Sci*	Complications in the Study of Ancient Tuberculosis: Presence of Environmental Bacteria in Human Archaeological Remains	Original article	Ancient DNA; authenticity of sequence data; Mycobacteria other than tuberculosis; *Mycobacterium tuberculosis* complex; polymerase chain reaction; tuberculosis
Neparáczki et al. (2011) *Acta Biologica Szegediensis*	Preliminary results from the paleomicrobiological studies of *Mycobacterium tuberculosis* infection in the Bácsalmás-Óalmás anthropological series	Original article	Paleopathology of tuberculosis; ancient DNA; Hungary; *Mycobacterium tuberculosis*
Posa et al. (2012) *Acta Biol Szeged*	Ancient Human Tooth Samples Used for TB Paleomicrobial Research	Original article	Paleopathology; paleomicrobiology; aDNA; skeletal tuberculosis; *Mycobacterium tuberculosis*; teeth
Posa et al. (2015) *Tuberculosis*	Tuberculosis in Late Neolithic-Early Copper Age human skeletal remains from Hungary	Original article	skeletal tuberculosis; late neolithic; human samples; aDNA; *Mycobacterium tuberculosis* complex; Carpathian basin
Roberts (2015) *Tuberculosis*	Old World Tuberculosis: Evidence from Human Remains with a Review of Current Research and Future Prospects	Review	Europe; diagnosis; limitations; aDNA analysis; diet; mobility; vitamin D; agriculture; urbanism

Redman et al. (2009) *Tuberculosis (Edinb)*	Mycocerosic Acid Biomarkers for the Diagnosis of Tuberculosis in the Coimbra Skeletal Collection	Original article	Archaeology; skeletons; tuberculosis; lipid biomarkers; mycocerosates
Salo et al. (1994) *Proc Natl Acad Sci U S A*	Identification of *Mycobacterium tuberculosis* DNA in a Pre-Columbian Peruvian Mummy	Original article	Not available
Spekker et al. (2023) *Tuberculosis (Edinb).*	Lumbosacral tuberculosis, a rare manifestation of Pott's disease - How identified human skeletons from the pre-antibiotic era can be used as reference cases to establish a palaeopathological diagnosis of tuberculosis.	Original article	Spinal tuberculosis; lumbosacral junction; pre-antibiotic era; identified human skeletons; palaeopathological diagnosis; macromorphological examination
Spigelman et al. (1993) *Int J Osteoarchaeol*	The Use of the Polymerase Chain Reaction (PCR) to Detect *Mycobacterium tuberculosis* in Ancient Skeletons	Original article	Polymerase chain reaction (PCR); DNA; *Mycobacterium tuberculosis*; ancient, bones
Spigelman et al. (2002) *Int J Osteoarchaeol*	Confirmation of the presence of *Mycobacterium tuberculosis* complex-specific DNA in three archaeological specimens	Original article	Ancient DNA; Mycobacterium tuberculosis; PCR
Sreevatsan et al. (1997) *Proc Natl Acad Sci U S A*	Restricted Structural Gene Polymorphism in the *Mycobacterium tuberculosis* Complex Indicates Evolutionarily Recent Global Dissemination	Original article	Not available
Szikossy et al. (2015) *Tuberculosis*	Two positive tuberculosis cases in the late Nigrovits family; 18th century; Vac; Hungary	Original article	*Mycobacterium tuberculosis*; Vac; Hungary; mummies; paleopathology; paleomicrobiology
Taylor et al. (2007) *Microbiology*	First report of *Mycobacterium bovis* DNA in human remains from the Iron Age	Original article	Not available
Taylor et al. (2010) *Int J Osteoarchaeol*	Ancient DNA (ADNA) Studies of Man and Microbes: General Similarities, Specific Differences	Original article	Ancient DNA; PCR; validation
Teschler-Nicola et al. (2015) *Tuberculosis*	The Early Mediaeval manorial estate of Gars/Thunau, Lower Austria: An enclave of endemic tuberculosis?	Original article	Early mediaeval Austria; demography; pathology; tuberculosis; DNA sequencing isotopes; diet;mobility
Thierry et al. (1990) *J Clin Microbiol*	Characterization of a *Mycobacterium tuberculosis* Insertion Sequence, IS6110, and Its Application in Diagnosis	Original article	Not available
Váradi et al. (2021) *Acta Biol Szeged*	Lipid Biomarker-Based Verifi Cation of TB Infection in Mother's and Daughter's Mummified Human Remains (Vác Mummy Collection, 18th Century, CE, Hungary)	Original article	High-performance liquid chromatography; lipid biomarkers; mass spectrometry; mummies; mycocerosates; tuberculosis
Vargová et al. (2017) *Tuberculosis*	Brief History of Tuberculosis in the Czech Lands	Review	Specific inflammation; skeletal remains; central Europe; *Mycobacterium tuberculosis*; paleopathology
Wilbur et al. (2009) *J Archaeol Sci*	Deficiencies and challenges in the study of ancient tuberculosis DNA	Review	Ancient DNA; biomolecular archaeology; paleopathology; polymerase chain reaction; spoligotyping; tuberculosis
Witas et al. (2015) *Eur J Clin Microbiol Infect Dis*	Molecular studies on ancient *M. tuberculosis* and *M. leprae*: methods of pathogen and host DNA analysis	Review	Not available

Wooding et al. (2019) *Int J Osteoarchaeol*	Reviewing the palaeopathological evidence for bovine tuberculosis in the associated bone groups at Wetwang Slack, East Yorkshire	Original article	ancient DNA; bovine tuberculosis; Britain, faunal remains; human osteology; human remains; Iron Age; *Mycobacterium bovis* (bTB); palaeopathology; zooarchaeology; zoonoses
Zink et al. (2001) J Med Microbiol	Molecular Analysis of Skeletal Tuberculosis in an Ancient Egyptian Population	Original article	Not available
Zink et al. (2003) *J Clin Microbiol*	Characterization of *Mycobacterium tuberculosis* Complex DNAs from Egyptian Mummies by Spoligotyping	Original article	Not available

The table details the main features of the included records: authors, type of study and keywords. Abbreviations: PPNB, Pre-pottery Neolithic B; NGS, next-generation sequencing; PCR, polymerase chain reaction; qPCR, quantitative polymerase chain reaction; aDNA, ancient DNA; IS, insertion sequences.
